# Selective Alpha-Particle Mediated Depletion of Tumor Vasculature with Vascular Normalization

**DOI:** 10.1371/journal.pone.0000267

**Published:** 2007-03-07

**Authors:** Jaspreet Singh Jaggi, Erik Henke, Surya V. Seshan, Barry J. Kappel, Debjit Chattopadhyay, Chad May, Michael R. McDevitt, Daniel Nolan, Vivek Mittal, Robert Benezra, David A. Scheinberg

**Affiliations:** 1 Molecular Pharmacology and Chemistry Program, Memorial Sloan-Kettering Cancer Center, New York, New York, United States of America; 2 Cancer Biology and Genetics Program, Memorial Sloan-Kettering Cancer Center, New York, New York, United States of America; 3 Department of Pathology, Weill Medical College of Cornell University, New York, New York, United States of America; 4 ImClone Systems Incorporated, New York, New York, United States of America; 5 Cold Spring Harbor Laboratory, Woodbury, New York, United States of America; Ordway Research Institute, Inc., United States of America

## Abstract

**Background:**

Abnormal regulation of angiogenesis in tumors results in the formation of vessels that are necessary for tumor growth, but compromised in structure and function. Abnormal tumor vasculature impairs oxygen and drug delivery and results in radiotherapy and chemotherapy resistance, respectively. Alpha particles are extraordinarily potent, short-ranged radiations with geometry uniquely suitable for selectively killing neovasculature.

**Methodology and Principal Findings:**

Actinium-225 (^225^Ac)-E4G10, an alpha-emitting antibody construct reactive with the unengaged form of vascular endothelial cadherin, is capable of potent, selective killing of tumor neovascular endothelium and late endothelial progenitors in bone-marrow and blood. No specific normal-tissue uptake of E4G10 was seen by imaging or post-mortem biodistribution studies in mice. In a mouse-model of prostatic carcinoma, ^225^Ac-E4G10 treatment resulted in inhibition of tumor growth, lower serum prostate specific antigen level and markedly prolonged survival, which was further enhanced by subsequent administration of paclitaxel. Immunohistochemistry revealed lower vessel density and enhanced tumor cell apoptosis in ^225^Ac-E4G10 treated tumors. Additionally, the residual tumor vasculature appeared normalized as evident by enhanced pericyte coverage following ^225^Ac-E4G10 therapy. However, no toxicity was observed in vascularized normal organs following ^225^Ac-E4G10 therapy.

**Conclusions:**

The data suggest that alpha-particle immunotherapy to neovasculature, alone or in combination with sequential chemotherapy, is an effective approach to cancer therapy.

## Introduction

Inhibition of tumor angiogenesis is an emerging treatment strategy for solid tumors [1]. Endothelium-targeting peptides, antibodies, antibody fragments and nanoparticles have been used to target the tumor vasculature in various preclinical and clinical studies [2], [3], [4]. The ultimate goal of these anti-angiogenic strategies is to inhibit endothelial cell proliferation in tumors via either targeted delivery of toxins, cytotoxic drugs or radiation to endothelial cells, interference with intercellular signaling pathways in endothelial cells (e.g. anti-VEGF therapies) [5], [6], [7], [8], [9] or disruption of endothelial cell interaction with the extracellular matrix (e.g. α_v_β_3_ integrin inhibitors) [10]. Endothelial cells, unlike cancer cells, are generally genetically and phenotypically stable and do not mutate readily; therefore, development of drug-resistance is not a major concern in therapies directed against endothelial cells [11].

Tumor growth inhibition via anti-angiogenic therapy has certain practical limitations to its implementation [12]. A second wave of angiogenesis initiated by the residual tumor cells can ensue when an anti-angiogenic treatment is discontinued, leading to a late resurgence in tumor growth [13], [14]. Therefore, a combination of anti-angiogenic therapy and cytotoxic therapy that targets the tumor cells directly has been suggested to prevent tumor recurrence. However, destruction of tumor vasculature following anti-angiogenic therapy can decrease blood flow to tumors and potentially prevent the delivery of anti-tumor therapeutics to the tumor cells [12]. Recently, Jain *et al* have shown that anti-angiogenic therapies may transiently increase the efficiency of the tumor vasculature, and that administration of cytotoxic therapy in that period may result in enhanced cytotoxic drug delivery to tumor cells [15]. Therefore, optimal scheduling of anti-angiogenic and chemotherapy may be required to overcome the pharmacokinetic barriers and could potentially result in long-term tumor remissions.

Vascular endothelial (VE) cadherin is a vascular endothelial cell specific molecule that is expressed constitutively throughout the entire vasculature and takes part in the formation of adherens junctions between adjacent endothelial cells [16]. It is required for the assembly of vascular structures during angiogenesis and maintenance of vascular integrity. The monoclonal antibody E4G10 specifically binds to an epitope exposed only on the monomeric, unengaged form of VE cadherin; the epitope gets masked on transdimerization to form inter-cellular junctions (Figure S1). This allows for selective targeting of endothelial cells in nascent tumor vasculature as well as of VE cadherin positive endothelial progenitor cells (EPCs) in bone marrow and peripheral circulation. Since E4G10 does not bind established vasculature, no vascular leak and hemorrhage is observed in normal organs of mice after E4G10 administration [17]. The described properties make E4G10 antibody an excellent targeting moiety for anti-angiogenic therapy.

Alpha particles are extraordinarily potent, short-ranged radiations with geometry uniquely suitable for selectively killing neovasculature. A single alpha particle track through the nucleus can kill a cell [18]. Therefore, we coupled E4G10 to chelated Actinium-225 (^225^Ac, an atomic-sized generator of an alpha particle-emitting isotope cascade [19], [20]), to produce an agent that could potently and selectively kill neovascular endothelium as well as the endothelial progenitors in the bone marrow and blood. Here we demonstrate, in a mouse model of prostatic carcinoma, the safety and efficacy of the ^225^Ac-E4G10 construct as a selective anti-angiogenic agent. Treatment with ^225^Ac-E4G10 suppressed tumor growth, enhanced tumor cell apoptosis and prolonged animal survival, without gross or histopathological toxicity in normal tissues or their vasculature. Synchronized administration of ^225^Ac-E4G10 and paclitaxel resulted in enhancement of the anti-tumor response.

## Methods

### Animals

Male BALB/c and athymic nude mice (NCr nu*/nu*), 4–12 weeks of age, were obtained from Taconic, Germantown, NY. All animal studies were conducted according to the NIH *Guide for the care and use of laboratory animals* and were approved by the Institutional Animal Care and Use committee at Memorial Sloan Kettering Cancer Center.

### Flow cytometry

Flow cytometric analysis of H5V and LNCaP cells was performed with anti-CD31 (Pharmingen, San Diego, CA), E4G10, J591 (anti-prostate specific membrane antigen) or isotype control antibodies (R&D systems, Minneapolis, MN) and fluorochrome-labeled secondary antibodies. Samples were acquired on an FC500 flow-cytometer (Beckman Coulter, Fullerton, CA) and analyzed with FlowJo software (Tree Star Inc., Ashland, OR).

### Preparation, quality control and administration of Radioimmunoconjugates

^225^Ac (Oak Ridge National Laboratory, Oak Ridge, TN) and Indium-111 (^111^In; Perkin Elmer, Boston, MA) were conjugated to E4G10 or non-specific rat IgG2a isotype antibody using a two-step labeling method, as described [21]. Routine quality control of the labeled antibody was performed using instant thin layer chromatography to estimate the radio-purity and cell binding assay to determine the immunoreactivity. Mice were anesthetized and then injected intravenously (in retro-orbital venous plexus) with the radioimmunoconjugate. The injected volume was 100µl and the antibody dose was 0.6–0.7 µg per 50 nCi injection. Typical radiochemical purity was 95–99%.

### Gamma Camera imaging and biodistribution

For gamma-imaging, anesthetized animals were imaged (in prone position) on X-SPECT™ scanner (Gamma Medica, Northridge, CA), a dedicated rodent imaging device, at specified time-points post-injection with 230µCi of ^111^In-E4G10. Images were acquired in a 56×56×16 image matrix using photopeak energy windows of 172 keV±10% and 273 keV±10% and no zoom. For organ distribution studies, mice were sacrificed at indicated time-points post-injection with ^111^In-E4G10 (3µCi) and their blood and the specified organs were harvested. The organs were washed in distilled water, blotted dry on gauze, weighed and the activity of ^111^In (15–550 keV window) was measured using a gamma-counter (COBRA II, Packard Instrument Company, Meriden, CT). Samples of the injectate (100µl) were used as decay correction standards. Percentage of injected dose of ^111^In per gram of tissue weight (%ID/g) was calculated for each animal and the mean %ID/g was determined at each time-point, as described previously [22], [23].

### Tumor implantation in mice

LNCaP prostate tumor cell line was obtained from the American Type Culture Collection (Rockville, MD). The LNCaP cells were grown in RPMI 1640 medium supplemented with L-glutamine, 10% fetal bovine serum and penicillin-streptomycin in an atmosphere of 5% CO2 and air at 37 degrees C. The cells were harvested and 1 million or 5 million cells were injected in 200µL matrigel (BD Biosciences, Palo Alto, CA) into the right flank of the animal. Animals were checked twice weekly for the development of palpable tumors at the site of injection.

### Tumor therapy studies

In the first ^225^Ac-E4G10 monotherapy study, mice were engrafted with 1 million LNCaP cells. The test group received 50 nCi of ^225^Ac labeled E4G10. Controls included vehicle (received 1% human serum albumin), unlabeled E4G10 (received 7 µg E4G10), ^225^Ac labeled isotype control (received 50 nCi [0.6 µg] of ^225^Ac labeled irrelevant rat IgG2a). Treatments were administered at 3, 5, 7 and 10 days post-implantation of xenografts. In the second ^225^Ac-E4G10 monotherapy study, mice were injected with 5 million LNCaP cells and treated on days 3, 5, 7 and 10 days post xenograft implantation with either vehicle (received 1% human serum albumin), 50 nCi of ^225^Ac labeled irrelevant isotype control IgG mixed with 7 µg of unlabeled specific E4G10 (dual control) or 50 nCi of ^225^Ac labeled E4G10. For the combination therapy study, ^225^Ac labeled E4G10 or isotype control antibody (50 nCi) was administered at 16, 18, 21 and 23 days post-implantation with 5 million LNCaP cells. Paclitaxel (20 mg/kg i.p.) was administered to the specified groups on days 27, 30, 34 and 37. Tumor size was measured with calipers, and tumor volume was calculated by the formula 0.52×*d_1_* ^2^×*d_2_*, where *d_1_* is the smaller diameter and *d*_2_ is the larger diameter. Animals were followed over long term for survival advantage. Mice were bled retro-orbitally on described days and serum prostate specific antigen (PSA) was determined using an immunoassay kit (Alpco diagnostics, Windham, NH).

### Histopathologic toxicity studies

BALB/c mice (n = 5) were injected four times with 100 nCi ^225^Ac-E4G10 (twice the dose at same schedule as the tumor therapy experiments). Animals were sacrificed 10 days after last injection and their lungs, kidneys, heart, liver and spleen were excised, fixed and examined by light microscopy.

### Anatomic Pathology and Immunohistology

Tumors or normal organs from mice were harvested, formalin-fixed and paraffin-embedded. Three micron sections were stained with hematoxylin and eosin (H&E), Periodic–acid Schiff (PAS) and Masson's trichrome, and evaluated with an Olympus BX45 light microscope, as described [24]. Eight micron tumor-sections were immunostained with goat anti-CD31 (Santa Cruz Biotechnology, Santa Cruz, CA) and mouse anti-smooth muscle actin (α-SMA; Sigma, St. Louis, MO) as primary antibodies, biotinylated secondary antibodies and streptavidin-fluorophores as tertiary reagents. Images were acquired on a Leica TCS SP2 AOBS confocal laser-scanning microscope. Apoptosis was detected in 8 µm tumor-sections using the TUNEL assay (In situ cell death detection kit; Roche). Immunoperoxidase staining was performed for von Willebrand factor and caspase-3, using rabbit anti-von Willebrand factor (vWF; Dako, Carpinteria, CA) and rabbit anti-cleaved caspase-3 (Cell signaling technology, Beverly, MA), and imaged on a Zeiss Axiovert 200M microscope. Acquired images were evaluated using ImageJ software (http://rsb.info.nih.gov/ij). For each of the four random fields (571×428um) of tumor sections stained with vWF, the number of pixels of positive staining was divided by the total number of pixels, and expressed as a percentage. The degree of apoptosis was estimated in each randomly selected field (1142×857um) by calculating the percentage of TUNEL positive cells out of the total number of cells (as measured by nuclear counterstaining).

### Electron Microscopy

Pieces of tumor tissue were fixed in 4% paraformaldehyde, post-fixed in 1% Osmium tetroxide and later embedded in epon. Ultra-thin sections (200–400 Å) were cut on nickel grids, stained with uranyl acetate and lead citrate and examined using a transmission electron microscope (Hitachi H-7500, Pleasanton, CA).

### Statistical analyses

Graphs were constructed using Prism (Graphpad software Inc., SanDiego, CA). Statistical comparisons between the experimental groups were performed by either the Student's t-test (two-group comparison) or one-way ANOVA with Bonferroni's multiple comparison post-hoc test (three-group comparison). All statistical comparisons were two sided and the level of statistical significance was set at p<0.05.

## Results

### E4G10 binds to cultured endothelial cells but not to established vasculature

**Figure 1:**
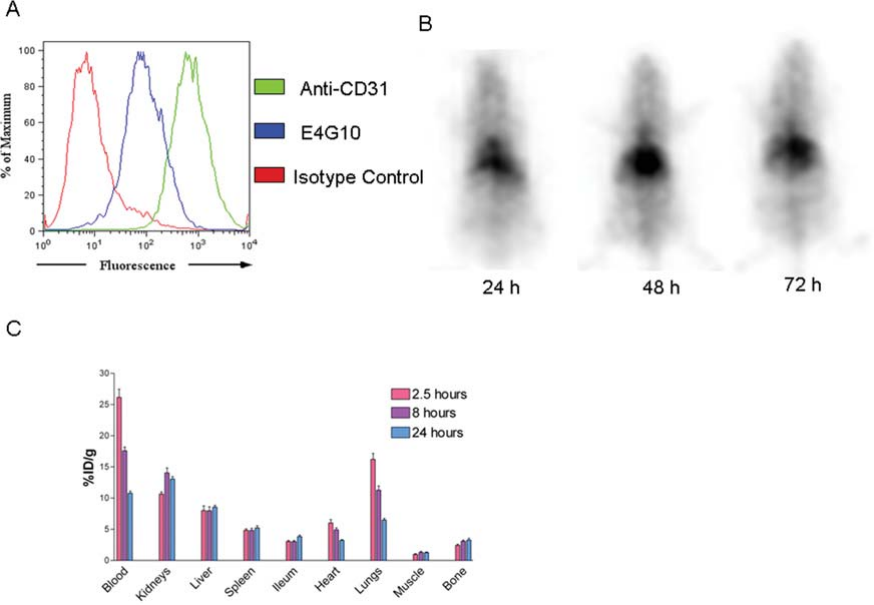
Characterization of E4G10. A, Flow cytometric analysis showing the binding of E4G10 to H5V cells, a mouse endothelioma cell line, in comparison to binding of the positive control anti-CD31 or isotype control antibody. B, X-SPECT gamma camera images of mice (prone, nose at top) at 24, 48 and 72 hours post-injection with ^111^In labeled E4G10. C, Biodistribution of ^111^In labeled E4G10 at specified time-points post-injection. Data are mean ± S.E.M. %ID/g = percentage of injected dose per gram of tissue.

The binding specificity of the monoclonal antibody E4G10 for endothelial cells of the neovasculature was determined *in vitro* and *in vivo* by its binding to H5V mouse endothelioma cells, and by the lack of specific uptake in normal tissues by imaging and biodistribution studies (Fig. 1). In flow cytometric studies, E4G10 bound with high affinity to H5V cells (Fig 1A). X-SPECT gamma camera images at various time-points post-injection with ^111^In trace-labeled E4G10 showed no organ-specific uptake of the radioactivity in BALB/c mice (Fig. 1B). The radioactivity gradually cleared from the blood pool and other vascularized organs such as heart and lungs. At later time-points, the radioactivity remained only at the sites of IgG catabolism such as the liver and spleen. Detailed quantitation of the biodistribution was performed by sacrificing animals at defined time-points post-injection with ^111^In-E4G10 and measuring the radioactivity in harvested organs (Fig. 1C). Therefore, the post-mortem data confirmed the lack of specific uptake of E4G10 in normal tissues seen in whole body imaging study.

### ^225^Ac-E4G10 inhibits the growth of prostate cancer xenografts in mice

**Figure 2:**
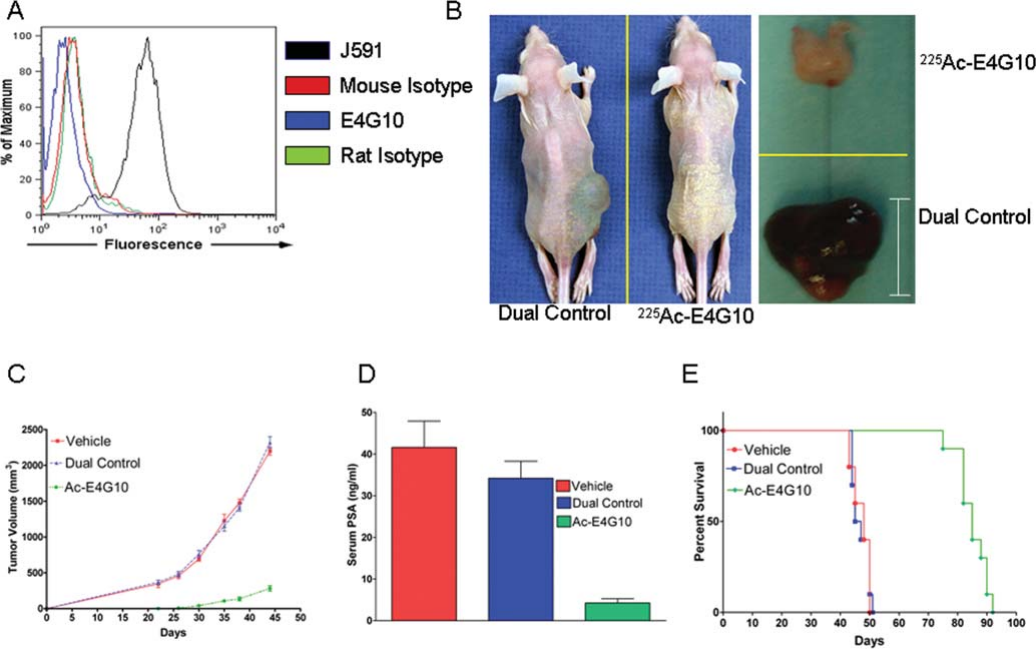
^225^Ac-E4G10 therapy inhibits the growth of LnCap prostate tumors. A, Flow cytometric analysis depicting the lack of E4G10 binding to LnCap cells; J591, mouse-anti prostate specific membrane antigen is the positive control. Mouse and rat isotype controls were also evaluated. B, Photographs of in situ (left) and excised tumor (right) in a representative dual control and ^225^Ac-E4G10 treated animal. C, Tumor volume in various treatment groups at described time-points. D, Serum prostate specific antigen (PSA) levels in the three treatment groups at 22 days post-implantation with 5 million LnCap cells. E, Kaplan Meier curve showing enhancement of survival with ^225^Ac-E4G10 treatment. Data in C, D are mean ± S.E.M. *Scale bar*, 1 cm.

E4G10 did not bind the human LNCaP prostate tumor cells in flow cytometric studies (Fig. 2A). The therapeutic efficacy of the ^225^Ac generator labeled E4G10 was tested in two separate experimental trials in athymic male mice that were xenografted with human LNCaP prostate tumors. ^225^Ac-E4G10 was therapeutically effective and significantly inhibited the growth of tumors. None of the control treatments had any significant effects on tumor growth (Fig. 2B & C; Fig. S2). Serum PSA, a surrogate marker for total body prostate tumor cell burden [25], was used to confirm the anti-tumor effects and was significantly lower ( p<0.001 vs. dual control; One way ANOVA and Bonferroni's post-hoc analysis) in ^225^Ac-E4G10 treated animals as compared to the controls (Fig. 2D). As a consequence of the anti-tumor effect, the median survival of ^225^Ac-E4G10 treated animals was longer relative to the control groups (Fig. 2E). Therefore, even though E4G10 did not bind to the LNCap tumors directly, treatment with ^225^Ac-labeled E4G10 resulted in an inhibition of tumor growth, lower serum PSA and enhanced survival in prostate cancer xenograft-bearing mice.

### Effects of ^225^Ac-E4G10 treatment on tumor histology

**Figure 3:**
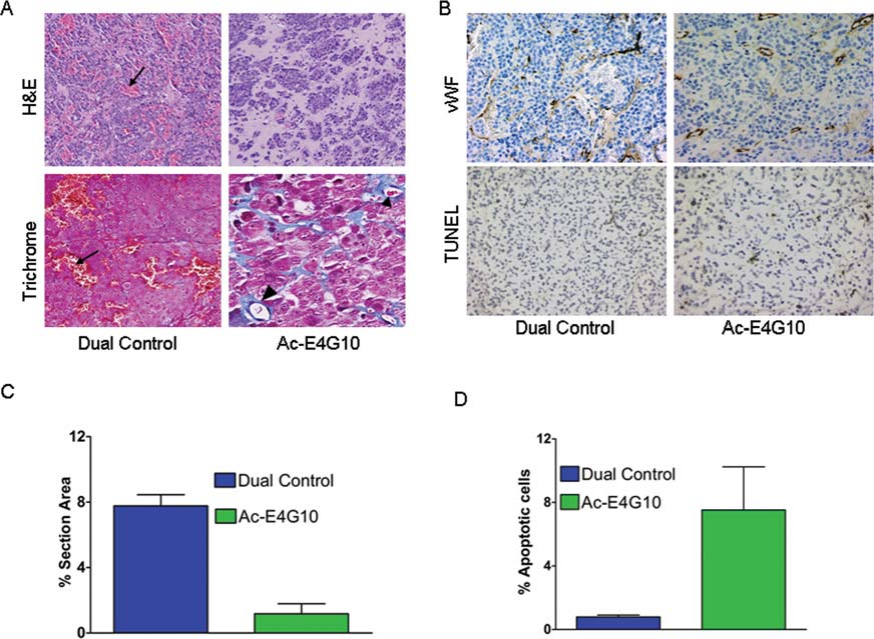
Effect of ^225^Ac-E4G10 therapy on tumor histology, vascularity and apoptosis. A, Light microscopy depicting numerous RBC-filled vascular spaces (arrows) in dual control tumor and fewer, but relatively normal-looking vessels (arrowheads) in the ^225^Ac-E4G10 treated tumor. B, Top: Immunohistochemical staining of tumor-sections for vWF, an endothelial cell marker (top). TUNEL staining of tumor sections to detect apoptosis (bottom). Quantification of vWF staining (C) and apoptosis (D) in 4 randomly selected fields. Data are mean ± S.E.M.

To dissect the mechanism of growth inhibition by ^225^Ac-E4G10, dual control and ^225^Ac-E4G10 treated animals were sacrificed at 14 and 22 days after tumor implantation (four animals per group at each time-point), and their tumors were excised and analyzed. The tumors in control animals were grossly hemorrhagic and on light microscopy, displayed infiltration of tumor cell masses by a network of markedly dilated, poorly defined, anastomosing vascular spaces filled with extravasated RBCs (Fig. 3A; Fig. S3). In contrast, ^225^Ac-E4G10 treated tumors showed groups of cohesive tumor cells separated by bands of acellular hyalinized stroma containing small, discrete and well-formed capillary vessels, which were lined by endothelial cells resting on a basement membrane (visualized with trichrome stain). Immunostaining for vWF, an endothelial cell marker, was significantly greater in the control tumors (p = 0.0002; Student's t-test) relative to the ^225^Ac-E4G10 treated ones (Fig. 3B & C). Additionally, TUNEL assay showed a significantly greater percentage of apoptotic cells in the ^225^Ac-E4G10 treated tumors (p = 0.0125; Student's t-test) relative to the control tumors (Fig. 3B & D). The TUNEL assay data was confirmed by cleaved caspase-3 immunohistochemistry (data not shown).

### ^225^Ac-E4G10 treatment leads to a relatively normalized tumor vasculature

**Figure 4:**
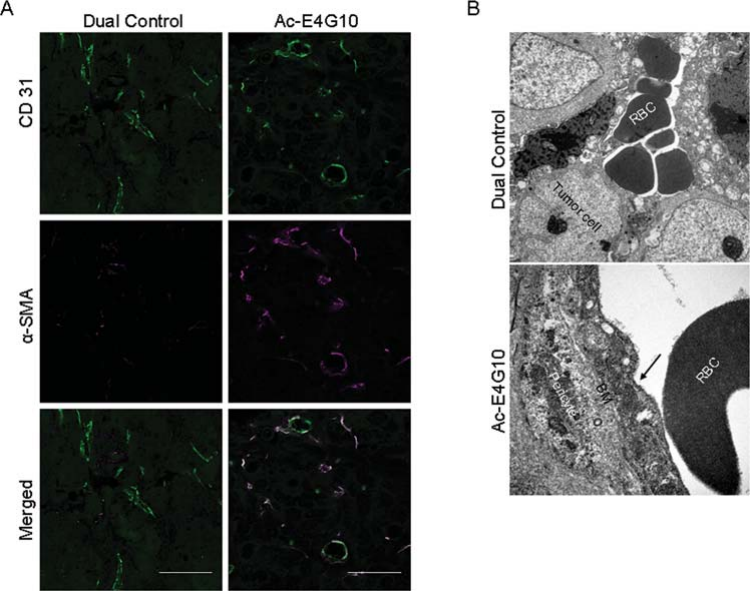
^225^Ac-E4G10 treatment results in a relatively normal remaining tumor vasculature. A, Greater coverage of tumor blood vessels (CD31 positive) by pericytes (α-SMA-positive cells) in ^225^Ac-E4G10 treated tumor relative to dual control. B, Transmission electron micrographs of blood vessels in dual control and ^225^Ac-E4G10 treated tumor. The dual control tumor contains extravasated RBC-filled vascular spaces that are not lined with endothelial cells, whereas blood vessels in ^225^Ac-E4G10 treated tumor display a continuous endothelial lining (arrow) resting on a basement membrane (BM) that is shared with the surrounding pericyte. Scale bar, 50 µm

To investigate whether treatment with ^225^Ac-E4G10, in addition to inhibiting tumor angiogenesis, also resulted in normalization of the residual tumor vasculature, tumor cross-sections were dual immunostained with CD31 (endothelial cell marker) and α-SMA (mural cell marker). The majority of the vascular endothelial cells in ^225^Ac-E4G10 treated tumor had pericyte coverage, whereas little coverage was observed in the tumor treated with the control agents (Fig. 4A & Movie S1). Transmission electron microscopy revealed sinusoid like blood vessels in dual control tumor, which were lined by tumor cells and filled with extravasated erythrocytes (RBCs, Fig. 4 B). In contrast, most vessels in ^225^Ac-E4G10 treated tumors appeared mature and were lined by a continuous layer of endothelial cells resting on a basement membrane and surrounded by a pericyte.

### Sequential administration of ^225^Ac-E4G10 and paclitaxel enhances the anti-tumor response

**Figure 5:**
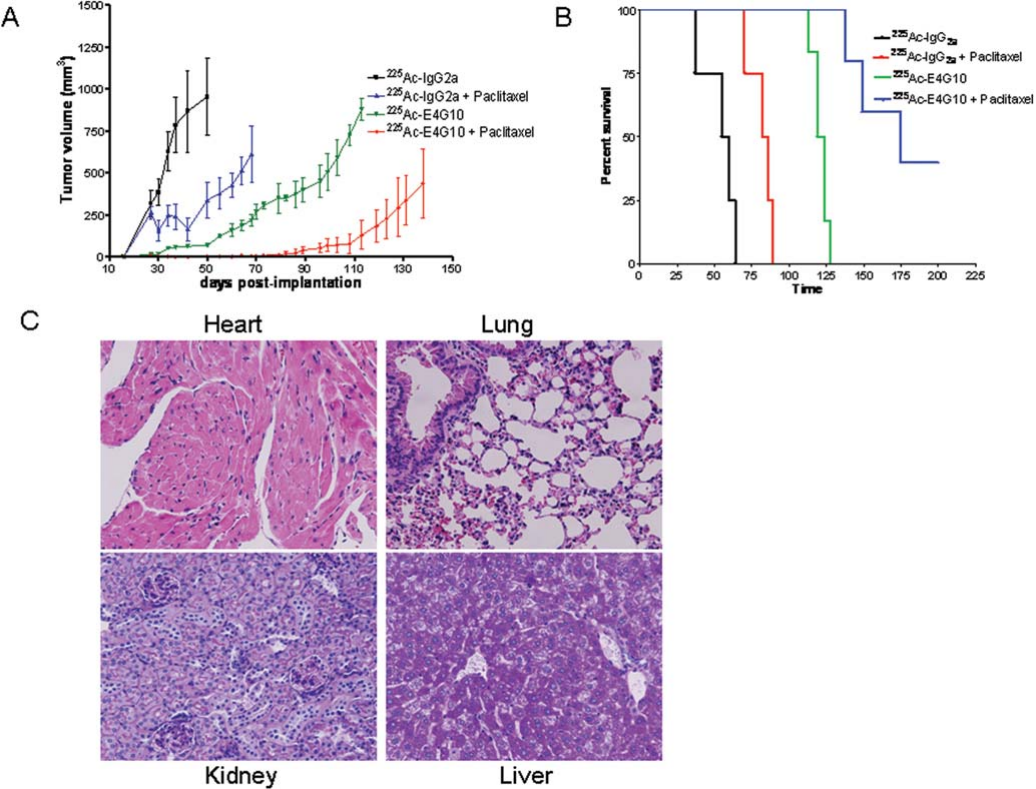
A combination of ^225^Ac-E4G10 with paclitaxel enhances the anti-tumor response. A, Tumor volume in the four treatment groups over time. Data are mean ± S.E.M. B, Kaplan Meier survival curve of treated animals showing significant enhancement of animal survival when ^225^Ac-E4G10 therapy is followed by a course of paclitaxel. C, Absence of histopathologic damage in normal organs, assessed 10 days after cessation of ^225^Ac-E4G10 treatment.

The structural normalization of residual tumor vasculature following ^225^Ac-E4G10 treatment prompted us to ask whether administration of a cytotoxic drug in that time-period would enhance the overall anti-tumor response via greater accessibility of the drug to tumor cells. Monotherapy with ^225^Ac-E4G10 significantly inhibited tumor growth and enhanced animal survival compared to controls as was observed in previous experiments (Fig. 5A & B). However, subsequent bi-weekly administration of paclitaxel for two weeks, starting four days after the last ^225^Ac-E4G10 injection resulted in a significant enhancement of the anti-tumor response compared to ^225^Ac-E4G10 monotherapy. Median survival for the specific combination treatment group was 182 days versus 113 days for the animals that received ^225^Ac-E4G10 alone or 84 days for animals that received ^225^Ac labeled isotype antibody and paclitaxel. Three animals each from the ^225^Ac-E4G10 and ^225^Ac-isotype control (IgG2a) group were sacrificed before commencement of paclitaxel therapy for histopathologic analyses of the tumor vasculature. As observed earlier (Fig. 3A), the tumor vasculature in ^225^Ac-E4G10 treated animals, though less extensive than that seen in ^225^Ac-isotype treated animals, displayed a relatively greater structural maturity (data not shown).

### ^225^Ac-E4G10 is not toxic at therapeutically active doses

The animals that received ^225^Ac labeled E4G10 or the isotype control antibody initially lost body weight (∼10%), which was recovered within 2 weeks. Histopathologic toxicity was studied in animals (n = 5) that received twice the dose of ^225^Ac-E4G10 at same schedule as in the therapy experiments. No evidence of vascular leakage or hemorrhage was observed in any of the examined normal organs (Fig. 5C). Additionally, the organs did not reveal any other gross or histopathologic abnormality.

## Discussion

We describe a novel cancer therapy of unusual potency and selectivity and elucidate its mechanism. Collectively, our data demonstrate the safety and effectiveness of specific delivery of short-ranged alpha particles to endothelial cells in inhibiting endothelial cell proliferation and tumor new vessel formation, resulting in suppression of tumor growth. Additionally, the treatment resulted in a relatively mature remaining tumor vasculature and an enhanced overall anti-tumor response when combined with subsequently administered chemotherapy.

Most anti-angiogenic therapies that are being developed or have been approved target cytokines, growth factors or their receptors. However, tumors may circumvent therapies aimed at a single signaling pathway via up-regulation of alternate pathways and therefore, targeting of multiple angiogenic pathways has been suggested [26], [27], [28]. Selective killing of endothelial cells in the tumor neovasculature or their progenitors with a cytotoxic agent is an attractive alternative approach to overcome the acquired resistance. We exploited the exclusive binding specificity of the antibody E4G10 for the endothelial cells of the neovasculature as well as VE Cadherin positive EPCs in the bone marrow and blood via its proposed targeting of an epitope exposed only on the monomeric, unengaged form of VE cadherin, which gets masked on the formation of adherens junctions between adjacent endothelial cells (Fig. S1). Therefore, based on the proposed mechanism, the antibody should not target established vasculature. Our in vivo imaging and post-mortem biodistribution data confirm the proposed selectivity of the antibody for neovasculature and EPCs. The uptake of radioactivity seen in the lungs and the heart was due to the presence of radiolabeled antibody in the blood pool in these organs and it declined in proportion to the blood clearance of radiolabeled antibody. As a consequence of selective targeting of a minor subpopulation of cells, in conjunction with the short range of the alpha particles, no discernible toxicity was seen in therapy studies with ^225^Ac-E4G10. Additionally, administration of supra-therapeutic doses of ^225^Ac-E4G10 in mice did not result in any histopathologic abnormality, vascular leak or hemorrhage in normal organs as has been seen with other VE-cadherin-binding antibodies that disrupt adherens junctions in established vessels [17]. This result further validated the pharmacokinetic data that E4G10 did not specifically accumulate in normal tissues with established blood vessels as the target VE-cadherin epitope for EG410 is masked in those vessels.

^225^Ac was considered as a suitable cytotoxic agent for coupling to E4G10 because of its four alpha particle emissions per decay of a ^225^Ac atom, which contributes to the enhanced the potency of ^225^Ac labeled constructs. ^225^Ac labeled antibodies have been shown to be safe and potent anti-tumor agents in mouse models of solid prostatic carcinoma, disseminated lymphoma, intra-peritoneal ovarian cancer and in a rat model of meningeal neuroblastoma [20], [21], [23], [29]. The high energy (5–8 MeV) and short path-length (50–80 µm) makes alpha particles the most appropriate form of radiation for targeting of individual endothelial cells [21].

Even though EG410 did not bind to the LNCaP cells, treatment with ^225^Ac labeled E4G10 resulted in an inhibition of tumor growth, lower serum PSA and enhanced survival in prostate cancer xenograft-bearing mice, accompanied by a decrease in tumor blood vessel density (as evidenced by vWF immunostaining). Although ^225^Ac-E4G10 inhibited tumor growth, it did not eradicate tumors when used as a single agent. The result is consistent with the vascular-targeting mechanism of action of ^225^Ac-E4G10. Since the tumor cells are not targeted, the residual tumor cells (as seen in Fig. 3a) can initiate a second phase of angiogenesis which results in a resurgence in tumor growth. Specific depletion of VE-cadherin positive endothelial cells of the tumor neovasculature with ^225^Ac labeled E4G10 is one explanation for the observed decrease in tumor blood vessel density. Recently, it has been shown that hypoxic stress can enhance the release of endothelial progenitors from the bone marrow and their recruitment and incorporation into tumor vasculature [30]. Selective alpha particle-mediated killing of the VE-cadherin positive late endothelial progenitors in the bone marrow or circulating endothelial progenitor cells in the blood stream (which are readily accessible to the radiolabeled antibody) is another mechanism via which ^225^Ac-E4G10 may have inhibited tumor angiogenesis. Our related manuscript (Nolan *et al,* submitted) describes, in a Lewis lung cancer model, the mobilization of VE cadherin positive EPCs from the bone-marrow into the peripheral circulation and their incorporation into tumor neovessels. Treatment with ^225^Ac-E4G10 resulted in a significant decrease in the bone-marrow derived endothelial cell progenitors in the tumor and a lower tumor vessel density. Another plausible contribution to the pronounced inhibition of tumor growth, besides direct cytotoxicity to tumor neovascular endothelial cells or their progenitors, can be from the local release of α-particle emitting daughters of ^225^Ac (francium-221, astatine-217 and bismuth-213[19] in the tumor microenvironment as a result of ^225^Ac decay following binding of ^225^Ac-E4G10 to VE cadherin positive endothelial cells in nascent tumor vasculature.

Histopathologic examination of control tumors revealed a network of dilated, anastomosing vascular spaces that formed between tumor cell nests and were filled with extravasated RBCs. Although these tumors displayed significantly greater staining for vWF as compared to ^225^Ac-E4G10 treated ones, most of these vascular channels were not lined by endothelial cells and therefore, did not stain for vWF. Moreover, most vWF positive structures in the control tumors did not possess a lumen and may represent endothelial sprouts growing into the tumor [31]. These findings are consistent with previous data on this tumor model [32], [33]. A transient breach in vessel wall integrity secondary to growth factor-driven active endothelial cell proliferation and sprouting may have resulted in the extravasation of RBCs and the resultant intra-tumoral hemorrhage [31].

Blood vessels in tumors are abnormal in structure (dilated and torturous with abnormal basement membrane and inadequate pericyte coverage) and function (hyperpermeable; [11]. Tumor vessel leakiness correlates closely with histologic tumor grade [34]. A functionally compromised vasculature also precludes efficient delivery of oxygen and chemotherapeutics to the tumors. Furthermore, tumor hypoxia makes cancer cells resistant to radiation damage [35]. Previous studies have shown that inhibition of VEGF signaling can “normalize” the blood vessels and therefore, overcome these pharmacokinetic barriers to drug and oxygen delivery [15], [35]. An interesting finding in our study was that treatment with ^225^Ac-E4G10, in addition to reducing tumor blood vessel density, also resulted in a structurally mature residual tumor vasculature wherein a greater proportion of vascular endothelial cells had pericyte coverage as compared to control tumors. This could be attributed to pruning of immature tumor vessels via killing of excess endothelial cells or EPCs by treatment with ^225^Ac-E4G10. Therefore, inhibition of abnormal endothelial cell proliferation and the resultant vessel leak may possibly be the reason for the relatively normal residual tumor vasculature seen in our studies. The role of pericyte coverage in inhibiting metastasis in a murine pancreatic cancer model has been shown recently [36]. An inverse correlation between pericyte coverage and hematogenous spread has also been observed in colorectal cancer patients [37]. The effects of ^225^Ac-E4G10 treatment on tumor metastasis and invasiveness are currently being investigated.

Combination therapy wherein ^225^Ac-E4G10 treatment was followed by a course of paclitaxel resulted in an enhancement of the overall anti-tumor response. One plausible explanation for that effect is that the structural normalization of tumor vasculature by ^225^Ac-E4G10 treatment resulted in increased efficiency of the vessels in delivering the chemotherapeutic to the tumor cells, thereby leading to synergy. Alternatively, the two treatments may also have an additive effect by killing two distinct populations of cells (endothelial and tumor cells). Paclitaxel has also been shown to possess anti-angiogenic properties [38] but recent data by Kerbel *et al* suggests that the cremophor-based paclitaxel formulation (which was used in our experiments) does not have a significant impact on the tumor vasculature or viability of circulating endothelial progenitors [39]. Nonetheless, killing of tumor cells by a cytotoxic agent can possibly decompress blood vessels in a tumor and therefore, increase blood flow. The exact mechanism of the enhanced overall response is currently being investigated.

Our results allude to the development of an integrative approach to cancer therapy wherein ^225^Ac-E4G10 therapy is precisely timed with chemotherapy or radiation to maximize the delivery of the chemotherapeutic and to improve radiation sensitivity of tumors. Delivering the two treatment modalities in a carefully planned temporal fashion can potentially result in a synergistic effect on tumor-cell killing. Importantly, our data suggest that targeting the tumor cells or their microenvironment may not be necessary to slow cancer growth if the angiogenic progenitors, a relatively small but possibly sensitive cell population, can be selectively depleted.

## Supporting Information

Figure S1 Schematic depicting the selectivity of E4G10 for the unengaged form of VE-cadherin molecule and the effect of 225Ac-E4G10 therapy on tumor vasculature.
(2.79 MB TIF)

Figure S2 225Ac-E4G10 therapy inhibits the growth of LnCap prostate tumors. A, Tumor volume in various treatment groups (n = 5) at 30 days post-implantation with 1 million LnCap cells in Matrigel. B, Serum prostate specific antigen (PSA) levels in the different treatment groups at 42 days post-implantation C, Kaplan Meier survival curve showing enhancement of survival with 225Ac-E4G10 treatment. Data in A, B are mean ± s.e.m. Asterisk, P<0.05 vs. [Ac-225] IgG2a isotype control (One way ANOVA and Bonferroni's post-hoc analysis).
(2.30 MB TIF)

Figure S3 Effect of 225Ac-E4G10 therapy on tumor histology at 14 days post-tumor implantation. Light microscopy revealing numerous dilated, anastomosing, RBC-filled vascular spaces in dual control tumor and fewer, but relatively normal-looking vessels in 225Ac-E4G10 treated tumor.
(5.03 MB TIF)

Movie S1 Treatment with 225Ac-E4G10 leads to relatively mature remaining tumour vasculature. Three dimensional reconstruction stack of serial Z-plane slices (z-stack) through the 225Ac-E4G10 treated tumor section, immunostained for endothelial cells (CD31, green) and pericytes (SMA, violet). The endothelial cells display significant pericyte coverage. Image dimensions, 140×140 µm; 72 sections in z-dimension; total thickness, 12 µm.
(6.94 MB AVI)
