# Supplementary figures and images for: Selective Alpha-Particle Mediated Depletion of Tumor Vasculature with Vascular Normalization
Source: PLoS One. 2007 Mar 7;2(3):e267. doi: 10.1371/journal.pone.0000267 (PMC1801076; doi:10.1371/journal.pone.0000267)

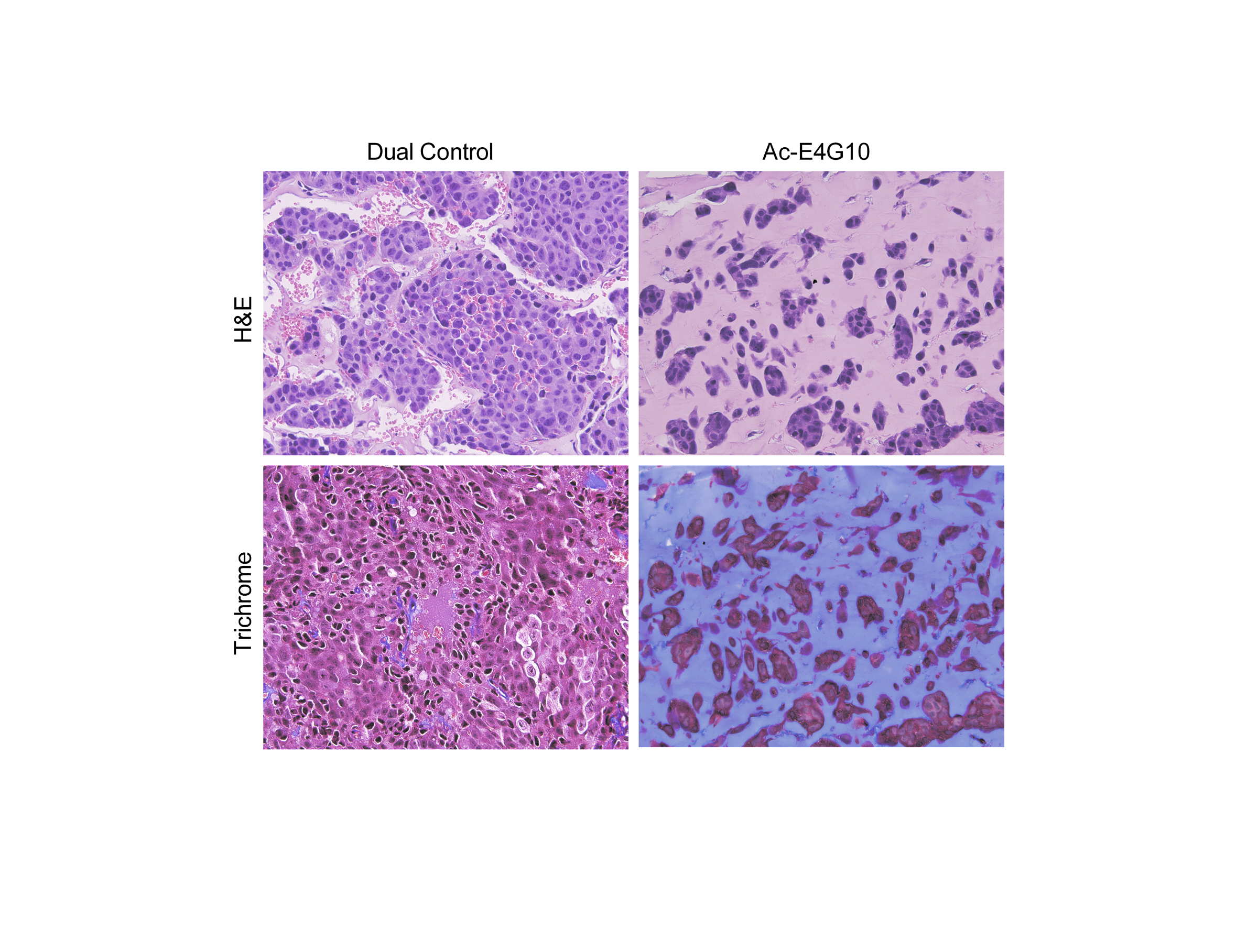

Supplement: Figure S3 — Effect of 225Ac-E4G10 therapy on tumor histology at 14 days post-tumor implantation. Light microscopy revealing numerous dilated, anastomosing, RBC-filled vascular spaces in dual control tumor and fewer, but relatively normal-looking vessels in 225Ac-E4G10 treated tumor. (5.03 MB TIF) [file pone.0000267.s003.tif]
